# The Association between Season of Pregnancy and Birth-Sex among Chinese

**DOI:** 10.3390/ijerph110808166

**Published:** 2014-08-11

**Authors:** Tan Xu, Dongdong Lin, Hui Liang, Mei Chen, Weijun Tong, Yongping Mu, Cindy Xin Feng, Yongqing Gao, Yumei Zheng, Wenjie Sun

**Affiliations:** 1 Department of Epidemiology, School of Public Health, Medical College of Soochow University, Suzhou, Jiangsu 215123, China; E-Mails: xutan@suda.edu.cn (T.X.); tongweijun.suda@live.com (W.T.); 2 Department of Epidemiology, School of Public Health and Tropical Medicine, Tulane University, New Orleans, LA 70112, USA; E-Mail: txu6@tulane.edu; 3 School of Science and Engineering, Tulane University, New Orleans, LA 70112, USA; E-Mail: dlin5@tulane.edu; 4 Zhejiang Provincial Center for Disease Control and Prevention, Hangzhou, Zhejiang 310051, China; E-Mail: hliang@cdc.zj; 5 Longquanyi District First Hospital, Chengdu, Sichuan 610100, China; E-Mail: drizzle-chen@163.com; 6 The Affiliated People’s Hospital of Inner Mongolia Medical University, Hohhot 010110, China; E-Mail: ypmu040@sina.com; 7 School of Public Health, University of Saskatchewan, Saskatoon SK S7N 5E5, Canada; E-Mail: cindy.feng@usask.ca; 8 School of Food Science, Guangdong Pharmaceutical University, Zhongshan 528458, China; E-Mail: yongqingg@163.com; 9 Center for Science Communication and Health Education Management, National Research Institute for Family Planning, Beijing 100081, China; 10 Department of Global Environmental Health Sciences, School of Public Health and Tropical Medicine, Tulane University, New Orleans, LA 70112, USA

**Keywords:** season, birth-sex, China

## Abstract

*Objective*: although numerous studies have reported the association between birth season and sex ratio, few studies have been conducted in subtropical regions in a non-Western setting. The present study assessed the effects of pregnancy season on birth sex ratio in China. *Methods*: We conducted a national population-based retrospective study from 2006–2008 with 3175 children-parents pairs enrolled in the Northeast regions of China. Demographics and data relating to pregnancy and birth were collected and analyzed. A multiple logistical regression model was fitted to estimate the regression coefficient and 95% confidence interval (CI) of refractive error for mother pregnancy season, adjusting for potential confounders. *Results*: After adjusting for parental age (cut-off point was 30 years), region, nationality, mother education level, and mother miscarriage history, there is a significant statistical different mother pregnancy season on birth-sex. Compared with mothers who were pregnant in spring, those pregnant in summer or winter had a high probability of delivering girls (*p* < 0.05). The birth-sex ratio varied with months. *Conclusions*: Our results suggested that mothers pregnant in summer and winter were more likely to deliver girls, compared with those pregnant in spring. Pregnancy season may play an important role in the birth-sex.

## 1. Introduction

Birth season has been associated with many human diseases [1] including cancer [2], or physiological activities e.g., sleep [3], and refractive error [4]. Data from mammals showed that natural factors could affect the birth-sex through the parental condition [5]. Birth-sex ratios of humans have been reported to be affected by climate-related risks, such as temperature [6], although the chromosomes determine the sex. The few studies that have examined the hypothesis that birth season and birth-sex are related have reported discrepant results, but have varied widely in design, analytical methods, age ranges and populations. For example, Rajani *et al.* collected the record of 8729 live births that occurred during the years 1973–1974 at an Indian hospital and found that from July to February, the live birth sex ratio decreased [7]. Limited by being a hospital-based study design without considering the potential confounders, these findings cannot withstand scrutiny. Using monthly data of 1959–2001, Melnikov *et al.* reported a highly significant seasonal pattern of the birth-sex ratio in western Siberia, with a peak of the boy ratio in summer and a trough in winter [8].

However, pregnancy season should be more essentially associated with birth-sex, although pregnancy season is associated with birth season. To our knowledge, no study has been conducted using a population-based cohort design to address the pregnancy season’s effect on the birth-sex. Hence, the pregnancy season-related effects on human birth-sex ratio thus remain poorly known and controversial. For example, Melnikov *et al.* suggested that just like in non-human mammals there was a breakdown of the “ovulatory” season in spring, there was a trough with birth-sex ratio (boy/girl) reversal during the “anovulatory” season in winter [8], while Jongbloet argued it could be more due to socioeconomic factors [9].

We tested the null hypothesis that there is no seasonal variation in birth-sex in the Northeast regions of China. We examined the association between pregnancy season and birth-sex ratio. Here, we use a simple epidemiological model to reveal multiannual predictability based on high-quality birth data for China, which has a population around 1.4 billion.

## 2. Methods

### 2.1. Participants

This research was designed to evaluate whether the season of pregnancy is associated with birth sex ratio in Inner Mongolia. To calculate the birth sex ratio accurately, the research area should be at least cover a population of 300,000, with a 10 per 1000 birth rate. A sample size of more than 3000 in one area can produce enough power to calculate a correct birth sex ratio. According to the administration areas in China, we used a multiple sage, randomizing and cluster sampling method to select Tongliao city in Inner Mongolia as a research field where Kerqin district, Baokang town and Shebaitu countryside were selected as study fields to represent city, county (or town) and rural population. Those selected areas, all children were investigated. Trained investigators collected and recorded the data with a standard questionnaire by using a face to face inquiry method. Data included was: (1) demographic characteristics; (2) any history of abortion and the medical history of the children’s mother, and in her pregnancy period; (3) delivery records. We recruited 3175 children-parents pairs. Two cases were missing data. We also excluded those not with abnormal birth dates, e.g., premature births. Finally, 3051 children who were born between 1 January 2006 to 31 November 2008 and their parents were investigated.

### 2.2. Statistic

SAS for Windows Statistical Software Package Version 8.2 (SAS Institute, Cary, NC, USA) was used for data processing and analysis. Birth-sex were analyzed as binary outcome variables and season of pregnancy was investigated as categorical variable, respectively. The seasons were categorized as spring (March, April, and May), summer (June, July, and August), fall (September, October, and November), and winter (December, January, and February) [4,10,11]. Spring was the reference group. The pregnancy season was calculated, according to the birth date (proceeding 280 days). Logistical regression models with pregnancy data were fitted to estimate the regression coefficients and 95% CIs of birth-sex for pregnancy season, adjusting for parental age (cut-off point was 30 years), region, nationality, mother’s education level, mother’s profession and mother’s miscarriage history. All the tests were two sided and the significance level was set at 0.05. The study was approved by the Ethics Committee, Soochow University and followed the tenets of the Declaration of Helsinki. Written informed consent was obtained from all parents after the nature of the study was explained.

## 3. Results

**Figure 1:**
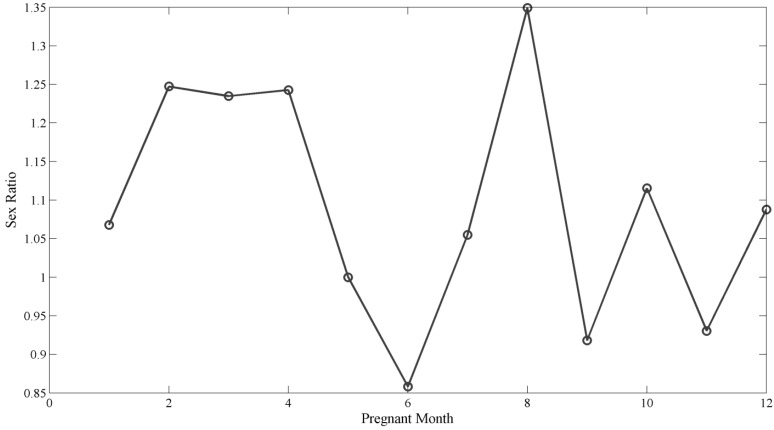
Birth-sex ratio (male/female) by mother pregnancy month.

Among 3051 children, there were 1582 boys (51.8%), and 1469 girls (48.2%). Table 1 shows the birth-sex characteristics. There are no statistical different between the boys and girls. After adjusting for parental age (cut-off point was 30 years), region, nationality, mother’s education level, mother profession, and mother’s miscarriage history, there is a significant statistical infuence between mother pregnancy season on birth-sex (*F*_13,3037_ = 1.94; *p* = 0.02). Table 2 shows that mothers pregnant in summer or winter had a higher probability of delivering girls (*p* < 0.05), than those pregnant in spring. The birth-sex ratio varied with months (Figure 1).

**Table 1:** Characteristics of the birth-sex.

Characteristic	Male	Female	Sex Ratio (M/F)	*p*-value
Father Age (Year)				
≥30	425	435	0.977	0.0999
<30	1157	1034	1.119	
Mother Age				
≥30	305	290	1.052	0.7825
<30	1277	1179	1.083	
Region				
City	1057	974	1.085	0.7946
Rural	525	495	1.061	
Pregnant Season				
Spring	386	311	1.241	0.0843
Summer	379	392	0.967	
Autumn	399	356	1.121	
Winter	418	410	1.020	
Nation				
Non-Mongol	828	754	1.098	0.6014
Mongol	754	715	1.055	
Mother education				
Junior high school	981	866	1.133	0.0910
High school	601	603	0.997	
Mother profession				
Famer	684	589	1.161	0.1263
Workers	250	278	0.899	
Civil servants	179	166	1.078	
Merchant	239	207	1.155	
Others	230	229	1.004	
Miscarriage history				
No	1522	1408	1.081	0.6774
Yes	60	61	0.984	

**Table 2:** Association of birth-sex and mother pregnancy season.

Characteristic	Odd Ratio	95% CI		*p*-value
Father Age (Year)				
≥30	Reference			
<30	0.815	0.65	1.02	0.075
Mother Age				
≥30	Reference			
<30	1.158	0.902	1.489	0.2516
Region				
City	Reference			
Rural	0.859	0.684	1.078	0.1912
Pregnant Season				
Spring	Reference			
Summer	0.782	0.636	0.961	0.0195 *
Autumn	0.912	0.74	1.123	0.3852
Winter	0.809	0.66	0.991	0.0412 *
Nation				
Non-Mongol	Reference			
Mongol	0.949	0.789	1.142	0.5796
Mother education				
Junior high school	Reference			
High school	0.859	0.71	1.039	0.1178
Mother profession				
Famer	Reference			
Workers	0.726	0.564	0.933	0.0124 *
Civil servants	0.981	0.716	1.346	0.9074
Merchant	0.983	0.759	1.273	0.8968
Others	0.852	0.664	1.092	0.206
Miscarriage history				
No	Reference			
Yes	0.920	0.633	1.336	0.6602

Notes: Adjusted for mother age, father age, region, nation, mother education level, mother profession and mother miscarriage history; * *p* < 0.05.

## 4. Discussion

Our results show that mothers who were pregnant in summer or winter are more likely to deliver girls than boys, compared with those pregnant in spring. Our results were partly aligned with previous studies and confirmed that there is a seasonality variation of birth-sex ratio [12]. Helle *et al.* examined the association between reconstructed annual mean temperatures and annual offspring sex ratio at birth in the eighteenth to nineteenth century Sami from northern Finland. Their results showed that warm years correlated with a male-biased sex ratio, whereas a warm previous year skewed sex ratio towards females. The net effect of one degree Celsius increase in mean temperature during these two years corresponded to approximately 1% more sons born annually [13]. However, the birth year’s temperature might be not related with birth-sex because gender is determined in the initial stage of the pregnancy. It is well known that the gender can be detected after 20 weeks of pregnancy. Hence, the birth season’s related factors cannot affect the gender. It is more likely to be a marker linked with gender. Compared with birth season, the pregnancy season provides a more reasonable explanation of the potential biological mechanism. Previous studies on climate change provide strong evidence that thermal fluctuations are critical for shaping biological outcomes [14]. The evidence from reptiles such as lizard [15], or turtle [16] show that the temperature could affect the gender of the next generation. Parallel examples have been reported in other animals, e.g., teleost fish [17], grayling (*Salmonidae*) [18], flounder [19], and sea bass [20]. Although there are no existing ecological models of development and sex determination that can fully explained those phenomena, the potential biological mechanism of temperature-dependent sex determination involves gonadal aromatase (cyp19a) [21], which plays a role in irreversibly converting androgens into estrogens, and the Dmrt1 [22], FoxL2, and Wnt4 genes [23].

Additionally, other season related climatic factors might also be associated with off-springs’ birth-sex. Evidence from mammals indicates that other climatic factors such as greater evaporation also influence the second sex ratio in dairy cattle [24]. Another plausible explanation comes from Jame *et al.*’s study, showing that temperature-dependent physiological mechanism(s) may override maternal ability to facultatively adjust offspring sex, and thus produce neutral or even maladaptive sex ratio variation.

Our study has special implications for public health considering global climate change which eventually could results in season changes, e.g., extension of the more extreme weather seasons. Thus could be a potential risk factor for the birth ratio structure. Studies with a larger sample size and different setting are thus warranted.

## 5. Limitations

First, the temperature used in the study was a monthly average, which cannot reflect daily variations. Second, due to a lack of accurate sex-determination time in pregnancy, the results might be inaccurate. However, the factors such as the seasonal climatic factors are quite similar and steady, especially since we used the monthly average temperature in the study. Third, children born early or later than 40 weeks can affect the results, although our large sample size could compensate for this. Fourth, local residents could be born in other areas, although our study’s participants are from a small city/town where the population is very steady.

## 6. Conclusions

Our results suggested that mothers pregnant in summer or winter were more likely to deliver girls than those pregnant in spring. Pregnancy season may play an important role in the birth-sex.
